# Immunotherapy-related adverse events in real-world patients with advanced non-small cell lung cancer on chemoimmunotherapy: a Spinnaker study sub-analysis

**DOI:** 10.3389/fonc.2023.1163768

**Published:** 2023-05-31

**Authors:** Shobana Anpalakhan, Prerana Huddar, Roya Behrouzi, Alessio Signori, Judith Cave, Charles Comins, Alessio Cortellini, Alfredo Addeo, Carles Escriu, Hayley McKenzie, Gloria Barone, Lisa Murray, David J. Pinato, Christian Ottensmeier, Sara Campos, Sethupathi Muthuramalingam, Samuel Chan, Fabio Gomes, Giuseppe L. Banna

**Affiliations:** ^1^ Portsmouth Hospitals University NHS Trust, Portsmouth, United Kingdom; ^2^ The Christie NHS Foundation Trust, Manchester, United Kingdom; ^3^ University of Genoa, Genoa, Italy; ^4^ University of Southampton, Southampton, United Kingdom; ^5^ Bristol Royal Infirmary, Bristol, United Kingdom; ^6^ Department of Surgery and Cancer, Imperial College London, London, United Kingdom; ^7^ Medical Oncology, Fondazione Policlinico Universitario Campus Bio-Medico, Roma, Italy; ^8^ University Hospital Geneva, Geneva, Switzerland; ^9^ The Clatterbridge Cancer Centre NHS Foundation Trust, Liverpool, United Kingdom; ^10^ University Hospitals of Northamptonshire, Northampton, United Kingdom; ^11^ Division of Oncology, Department of Translational Medicine, University of Piemonte Orientale, Novara, Italy; ^12^ The Clatterbridge Cancer Centre NHS Foundation Trust, University of Liverpool, Liverpool, United Kingdom

**Keywords:** lung cancer, immunotherapy, immune-related adverse effects, neutrophil-to-lymphocyte ratio (NLR), systemic immune-inflammation index (SII), overall survival, non-small cell lung cancer, progression free survival

## Abstract

**Background:**

The Spinnaker study evaluated survival outcomes and prognostic factors in patients with advanced non-small-cell lung cancer receiving first-line chemoimmunotherapy in the real world. This sub-analysis assessed the immunotherapy-related adverse effects (irAEs) seen in this cohort, their impact on overall survival (OS) and progression-free survival (PFS), and related clinical factors.

**Methods:**

The Spinnaker study was a retrospective multicentre observational cohort study of patients treated with first-line pembrolizumab plus platinum-based chemotherapy in six United Kingdom and one Swiss oncology centres. Data were collected on patient characteristics, survival outcomes, frequency and severity of irAEs, and peripheral immune-inflammatory blood markers, including the neutrophil-to-lymphocyte ratio (NLR) and systemic immune-inflammation index (SII).

**Results:**

A total of 308 patients were included; 132 (43%) experienced any grade irAE, 100 (32%) Grade 1–2, and 49 (16%) Grade 3–4 irAEs. The median OS in patients with any grade irAES was significantly longer (17.5 months [95% CI, 13.4–21.6 months]) than those without (10.1 months [95% CI, 8.3–12.0 months]) (p<0.001), either if Grade 1–2 (p=0.003) or Grade 3–4 irAEs (p=0.042). The median PFS in patients with any grade irAEs was significantly longer (10.1 months [95% CI, 9.0–11.2 months]) than those without (6.1 months [95% CI, 5.2–7.1 months]) (p<0.001), either if Grade 1–2 (p=0.011) or Grade 3–4 irAEs (p=0.036). A higher rate of irAEs of any grade and specifically Grade 1–2 irAEs correlated with NLR <4 (p=0.013 and p=0.018), SII <1,440 (p=0.029 ad p=0.039), response to treatment (p=0.001 and p=0.034), a higher rate of treatment discontinuation (p<0.00001 and p=0.041), and the NHS-Lung prognostic classes (p=0.002 and p=0.008).

**Conclusions:**

These results confirm survival outcome benefits in patients with irAEs and suggest a higher likelihood of Grade 1–2 irAEs in patients with lower NLR or SII values or according to the NHS-Lung score.

## Background

Lung cancer is the leading cause of cancer-related mortality worldwide, with most cases being non-small-cell lung cancer (NSCLC) (1, 2). The pharmacological management of patients with NSCLC has had major advancements as a result of the immunotherapy options now available (3–6). One such option is pembrolizumab, a programmed death-1 (PD-1) inhibitor. Its use alongside chemotherapy in patients with advanced NSCLC regardless of programmed death-ligand 1 (PD-L1) status has demonstrated improved survival outcomes and is now the standard of care (7).

With these immunotherapeutic options come a multitude of immunotherapy-related adverse effects (irAEs) affecting various bodily systems (3–6). However, previous analyses have reported that patients with irAEs tended to have better survival outcomes than patients without irAEs, but these were observed in trial cohorts (8). A retrospective study of patients with advanced NSCLC on immunotherapy alone also concluded that improved survival outcomes were seen among patients with irAEs. Another study of patients either on chemoimmunotherapy or immunotherapy alone at a German centre found that patients with irAEs survived longer though (9). However, data within a real-world cohort of patients who are solely on combined chemoimmunotherapy for their advanced NSCLC has yet to be presented.

The retrospective Spinnaker study assessed the efficacy of chemoimmunotherapy in patients with advanced NSCLC and subsequently established the NHS-Lung score as a tool to inform prognostic information in these patients (10). This score consisted of the following factors: a high number of metastatic sites, squamous histology of the tumour, and a high systemic immune-inflammatory index (SII). The present analysis following on from the Spinnaker study aims to assess the irAEs seen in this real-world patient cohort, the frequency and severity of these irAEs, and their impact on survival outcomes, and identify related clinical factors.

## Materials and methods

The Spinnaker study was a retrospective multicentre cohort study, which included patients with histologically confirmed advanced NSCLC, no actionable genetic alterations, and any PD-L1 tumour proportion score (TPS). These patients were of Eastern Cooperative Oncology Group Performance Status (ECOG PS) ≤ 1. They received first-line chemotherapy alongside pembrolizumab at one of seven different centres (six in the United Kingdom and one in Switzerland) between March 2018 and April 2021 (10).

Data were collected on patient characteristics, tumour characteristics, survival outcomes, disease response, frequency and severity of irAEs, treatment discontinuation rates, and peripheral immune-inflammatory blood markers such as the neutrophil-to-lymphocyte ratio (NLR) and SII. NLR was derived from the ratio of the number of neutrophils to the number of lymphocytes measured from a blood count check of a peripheral blood test taken within 14 days of the treatment start date. A high NLR was defined as ≥4 as previously reported (11). The SII was calculated from the product of the NLR and the platelet count, with the cut-off threshold being ≥1,440 (12). The definition of irAEs was based on the causality established by the responsible physician in each participating centre between the AE and immunotherapy. The severity of the irAEs was agreed on by clinical judgement and graded referring to the common toxicity criteria (CTC)-AE version 5. In the subgroup analysis, patients who developed an irAE that started as Grade 1–2 before progressing to Grade 3–4 were counted as a single case of irAE of any grade.

The primary endpoint of this analysis was to describe the frequency, type, and severity of irAEs observed in these patients and how these affected their survival outcomes (i.e., overall survival (OS) and progression-free survival (PFS)). Secondary endpoints included assessing for possible clinical factors influencing the likelihood of irAEs.

Clinical data were analysed by descriptive statistics using percentages for binary variables and medians for continuous variables, with their respective dispersion values reported. The chi-square test was used when comparing binary variables, and a significance value of p<0.05 was defined. The OS was calculated from the treatment start date until death or the date of the last follow-up, and the PFS was calculated from the treatment start date to disease progression or death from any cause. Patients who had not had any events at the time of the analysis were censored. OS and PFS were estimated using the Kaplan–Meier method and reported as medians with 95% confidence limits (95% CI) and compared using a two-sided log-rank test with an acceptable significance value of p<0.05 (13). A Spearman correlation test was performed between the irAE subgroups (i.e., any grade, Grade 1–2, and Grade 3–4) and various patients and tumour and blood marker prognostic factors. We performed an exploratory Cox regression analysis according to the irAEs. As more than one organ toxicity may occur in the same patient, we first assessed the role of single versus multiple organ irAEs, then according to the type of single organ irAEs occurring in at least 10 patients. The statistical analysis was carried out by SigmaPlot software version 12.5 (Systat Software, San Jose, CA).

This study was registered and approved as an audit by the multiple participating sites, with the coordinating centre being Portsmouth Hospital University NHS Trust (United Kingdom). Clinical data were anonymised before sharing with the coordinating centre for analysis. The audit procedures were compliant with the Data Protection Act 2018, the precepts of Good Clinical Practice guidelines with regard to the collection, storage, processing, and disclosure of personal information, and the principles outlined in the Declaration of Helsinki for all human or animal experimental investigations.

## Results

### Frequency, type, and severity of irAEs

The Spinnaker study included 308 patients from seven different centres (10). The characteristics of this patient cohort are described in **Table 1**. The median follow-up duration was 18 months (15.0–20.1 months). There were 132 cases of irAEs of any grade (43% of patients). One hundred patients (32%) developed Grade 1–2 irAEs, and 49 patients (16%) had Grade 3–4 irAEs. **Table 2** describes the range of irAEs seen and their frequency. The three most common bodily systems affected by irAEs of any grade were the skin (12%), bowel (7%), and thyroid (7%). This distribution was similar for Grade 1–2 irAEs. The most frequently seen Grade 3–4 irAEs were colitis and pneumonitis (5% each) and hepatitis and skin toxicity (2% each). A total of 72 patients (23%) discontinued treatment due to toxicity.

**Table 1:** Patient characteristics and outcomes.

Characteristic	No. (%) [range]
Age	
** Median**	65 [37–84]
** ≥70 years**	98 (32)
Gender	
** Male**	171 (56)
** Female**	137 (44)
Smoking history	
** Never**	25 (8)
** Former**	192 (62)
** Current**	91 (30)
Histology	
** Squamous**	51 (17)
** Adenocarcinoma**	246 (80)
** Other^a^**	11 (3)
ECOG PS	
** 0**	127 (41)
** 1**	181 (59)
Stage	
** IIIB/IVA**	24 (8)
** IVA**	113 (37)
** IVB**	171 (56)
**BMI^b^**	
** Median**	24.8 [15.0–43.9]
** Underweight/normal**	16 (5)/146 (47)
** Overweight/obese**	100 (32)/46 (15)
Number of metastatic sites	
** ≥ 3**	103 (33)
**Brain metastases**	31 (10)
**Liver metastases**	37 (12)
PD-L1 IHC Ab^c^	
** 22C3/SP263**	145 (49)/151 (51)
** Negative**	165 (56)
** Positive**	111 (37)
** High**	20 (7)
** N/A**	12 (4)
**Oncogene (EGFR/ALK/ROS1)**	3 (1)
**Pre-treatment steroids**	33 (11)
**NLR ≥4**	164 (53)
**SII ≥ 1,444**	154 (50)
Type of chemotherapy	
** Cisplatin-Pemetrexed**	24 (8)
** Carboplatin-Pemetrexed**	240 (78)
** Carboplatin-Paclitaxel**	44 (14)
Best response^d^	
** CR**	2 (1)
** PR**	197 (67)
** SD**	52 (18)
** PD**	45 (15)
** N/A**	12 (2)
**GCSF given**	59 (19)
irAE	
** Any grade**	132 (43)
** G1–G2**	100 (32)
** G3–G4**	49 (16)
**Treatment discontinuation**^e^	72 (23)
**Median follow up (months) [95% CI]**	18.0 [15.9–20.1]
**Median OS (months) [95% CI]**	12.7 [10.2–15.2]
**Median PFS (months) [95% CI]**	8.0 [7.1–8.8]

Ab, antibody; BMI, body mass index; CI, confidence interval; CR, complete response; ECOG PS, Eastern Cooperative Oncology Group Performance Status; GCSF, granulocyte colony-stimulating factor; IHC, immunohistochemistry; mo., months; NA, not assessable; NLR, neutrophil-to-lymphocyte ratio; No. Number; OS, overall survival; PD-L1, programmed cell death-ligand-1; PD, progressive disease; PFS, progression-free survival; PR, partial response; SD, stable disease; SII, systemic immune-inflammatory index; TPS, tumour proportion score; yr, year.

a Including poorly differentiated (No. 6), undifferentiated (No. 2), sarcomatoid (No. 1), adenosquamous (No. 1), and pleomorphic (No. 1) histology.

b BMI was calculated using the formula of weight/height^2^ (kilograms/square metres) and categorized according to the World Health Organization (WHO) categories: underweight (BMI<18.5), normal weight (18.5≤BMI ≤ 24.9), overweight (25≤BMI ≤ 29.9), obese (BMI≥30).

c Negative, TPS >1%; positive, TPS 1-49%; high, TPS ≥ 50%.

d By RECIST version 1.1 criteria.

e Due to toxicity.

**Table 2:** Immunotherapy-related adverse effects.

irAE, No. (%)	Any grade No (%)	G1–G2 No (%)	G3–G4 No (%)
**Any irAE**	132 (43)	100 (32)	49 (16)
**Skin**	44 (14)	37 (12)	7 (2)
**Colitis**^a^	37 (12)	21 (7)	16 (5)
**Thyroid**	22 (7)	21 (7)	1 (0)
**Pneumonitis**	25 (8)	11 (4)	14 (5)
**Liver**^b^	16 (5)	9 (3)	7 (2)
**Nephritis**^b^	9 (3)	7 (2)	2 (1)
**Hypophysitis**	7 (2)	5 (2)	2 (1)
**Arthritis**	4 (1)	3 (1)	1 (0)
**Adrenal**	2 (1)	1 (0)	1 (0)
**Myasthenia**	1 (0)	0 (0)	1 (0)

a Only one toxic death in the whole series attributed to immunotherapy-related colitis.

b Includes both laboratory abnormalities and diagnosis.

### Association of irAEs with survival outcomes

**Figure 1:**
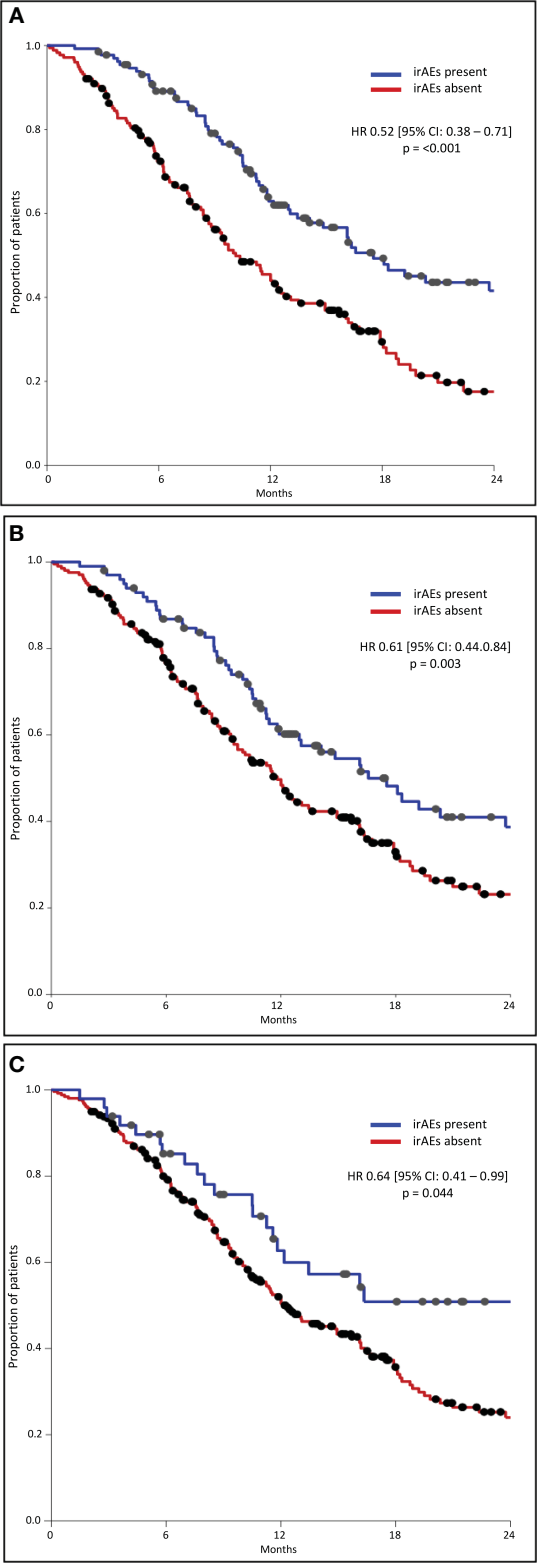
Overall survival – any grade **(A)**, G1-2 **(B)**, G3-4 **(C)**.

The median OS was significantly longer in all three subgroups of patients with any grade, Grade 1–2, and Grade 3–4 irAEs than in patients without irAEs (**Table 3**, **Figure 1**). Patients with irAEs of any grade had a median OS of 17.5 months (95% CI, 13.4–21.6 months), while patients without these irAEs had a median OS of 10.1 months (95% CI, 8.3–12.0 months) (p<0.001). Patients experiencing Grade 1–2 irAEs had a significantly longer median OS of 16.6 months (95% CI, 12.6–20.6 months) compared to those without who had a median OS of 11.8 months (95% CI, 10.1–13.6 months) (p=0.003). Patients experiencing Grade 3–4 irAEs also had a significantly longer median OS of 24.0 months (95% CI, 9.0–39.1 months) compared to those without who had a median OS of 12.1 months (95% CI, 9.8–14.5 months) (p=0.042).

**Table 3:** Overall survival according to grade of immunotherapy-related adverse effects.

irAE		No.	Median [95% confidence interval]	p-value
Any irAE	No	176	10.1 [8.3–12.0]	p < 0.001
	Yes	132	17.5 [13.4–21.6]	
G1**–**2 irAE	No	205	11.8 [10.1–13.6]	p = 0.003
	Yes	100	16.6 [12.6–20.6]	
G3**–**4 irAE	No	256	12.1 [9.8–14.5]	p = 0.042
	Yes	49	24.0 [9.0–39.1]	

**Figure 2:**
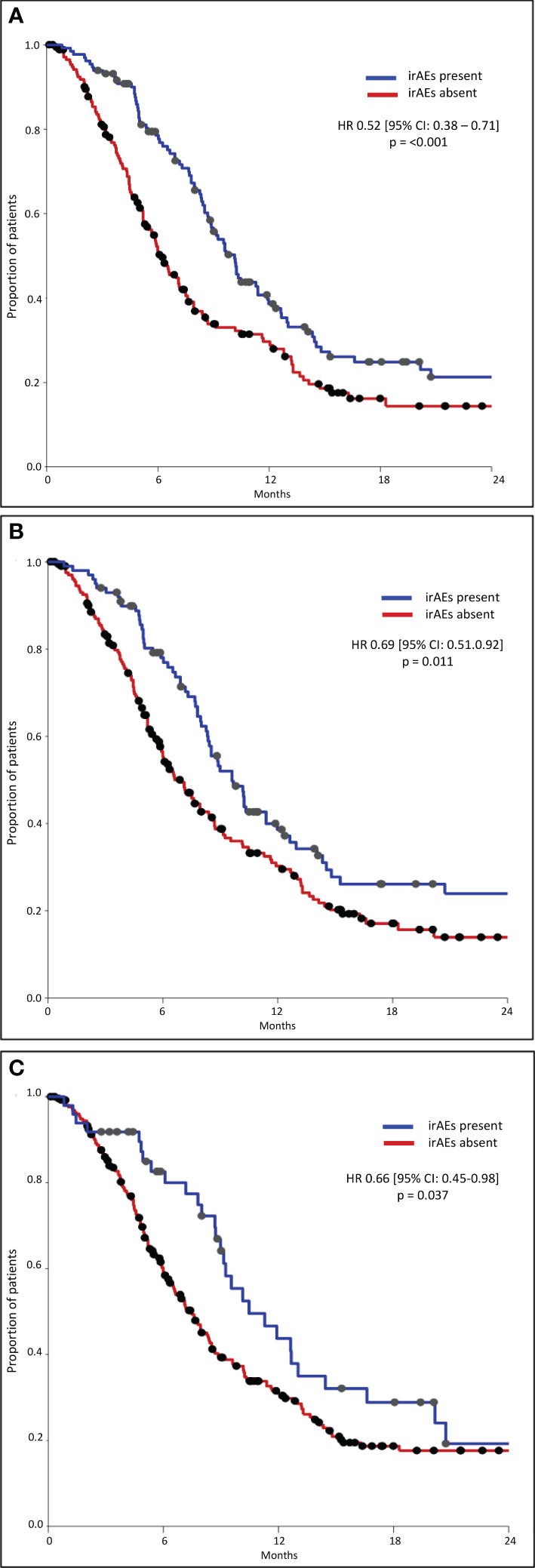
Progression-free survival – any grade **(A)**, G1-2 **(B)**, G3-4 **(C)**.

The median PFS was significantly longer in all three subgroups of patients with any grade, Grade 1–2, and Grade 3–4 irAEs than in patients without irAEs (**Table 4**, **Figure 2**). Patients with irAEs of any grade had a median PFS of 10.1 months (95% CI, 9.0–11.2 months), while patients without these irAEs had a median PFS of 6.1 months (95% CI, 5.2–7.1 months) (p<0.001). Patients experiencing Grade 1–2 irAEs had a significantly longer median PFS of 9.6 months (95% CI, 8.1–11.1 months) compared to those without who had a median PFS of 7.0 months (95% CI, 5.9–8.1 months) (p=0.011). Patients experiencing Grade 3–4 irAEs also had a significantly longer median PFS of 10.5 months (95% CI, 7.2–13.7 months) compared to those without who had a median PFS of 7.5 months (95% CI, 6.4–8.5 months) (p=0.036).

**Table 4:** Progression-free survival according to grade of immunotherapy-related adverse effects.

irAE		No.	Median [95% confidence interval]	p-value
Any irAE	No	176	6.1 [5.2–7.1]	p < 0.001
	Yes	132	10.1 [9.0–11.2]	
G1**–**2 irAE	No	205	7.0 [5.9–8.1]	p = 0.011
	Yes	100	9.6 [8.1–11.1]	
G3**–**4 irAE	No	256	7.5 [6.4–8.5]	p = 0.036
	Yes	49	10.5 [7.2–13.7]	

### Correlation of irAE with clinical prognostic factors

In the groups of patients with irAEs of any grade and those specifically with irAEs of Grade 1–2, a higher frequency of irAEs occurred in patients with NLR <4 (p=0.013 and p=0.018, respectively), SII <1,440 (p=0.029 and p=0.039, respectively), lower NHS-Lung score (p=0.002 and p=0.008, respectively), better disease response (p=0.001 and p=0.034, respectively) and if their treatment had been discontinued (p<0.00001 and p=0.041, respectively). In patients with Grade 3–4 irAEs, however, a higher frequency of irAEs was observed only in patients with better disease response (p=0.039) and if treatment had been discontinued (p=0.0001). There were no associations detected between the occurrence of irAEs and gender, pre-treatment PS, or PDL1 TPS.

### Association of irAEs type with survival outcomes

Both single and multiple organ irAEs were significantly associated with longer OS (p<0.001 and p=0.032, respectively), whereas only single-organ irAEs were significantly associated with longer PFS (p=0.002 and p=0.056). Within the limits of a non-landmark analysis, among single-organ irAEs, thyroid irAEs were significantly associated with both longer OS and PFS (p=0.009 and p=0.032), whereas skin irAEs were associated with longer OS (p=0.032) but not PFS (p=0.066) (**Supplementary Table S3**, **S4**).

## Discussion

The results of this analysis have shown that patients with irAEs of any grade had better survival outcomes regardless of the grade of the irAE. Potential predictors for the development of Grade 1–2 irAEs have also been identified including the NLR, the SII, the NHS-Lung score, disease response, and treatment discontinuation. Grade 3–4 irAEs were predicted only by disease response and treatment discontinuation. A possible explanation for the lack of correlation observed between Grade 3–4 irAEs and the other factors listed above could be due to the relatively low incidence of Grade 3–4 irAEs. The main limitations of this analysis include its retrospective nature and the lack of information on the timing of the irAEs observed. Nevertheless, the Spinnaker study was a multicentre project with a real-life cohort of patients lending itself to the generalisability of the results of this present analysis.

A previous pooled analysis of the IMpower130, IMpower132, and IMpower150 trials mirrored the findings from this analysis. It noted that patients with advanced NSCLC on a combination of chemotherapy, atezolizumab, and/or bevacizumab who experienced irAEs had longer OS compared to those without (8). This was also reflected in the outcomes of retrospective studies of patients with NSCLC who had received immunotherapy (9, 14). A previous work has shown that concurrent GCSF prophylaxis use in a proportion of patients in this cohort had no confounding impact on survival outcomes (15).

A systematic review of 51 studies assessing the use of immunotherapy in various solid malignancies, including lung cancers, detected a positive association between the development of irAEs and survival outcomes (16). Other works have echoed these findings among a variety of tumours being treated with immunotherapy (17–20). However, this present analysis highlights these associations between the presence of irAEs and improved survival outcomes among patients being treated with combined chemoimmunotherapy.

The results reported could be explained by a higher efficacy of immunotherapy in patients experiencing irAEs, thus conferring improved survival outcomes but potentially more irAEs. Therefore, the presence of irAEs could serve as a useful indicator for treatment response and survival. The potential for an immortal bias is also recognized, with possibly more irAEs being detected in patients surviving longer. A landmark analysis or a time-dependent Cox analysis could not be performed as the time of the immunotherapy-related adverse event was not recorded. Notably, in the present analysis, patients with irAEs had a higher rate of treatment discontinuation potentially indicating a low impact of the length of immunotherapy treatment on the improved survival outcomes.

There have been a number of reports suggesting potential predictive markers to identify patients who are more likely to develop irAEs. A prospective cohort study of patients with a solid or haematological malignancy in a French cancer centre found severe irAEs in those who had a PS ≥2 (21). All patients in the Spinnaker study were of PS 0–1, and therefore, a similar comparison to PS ≥2 cannot be made. However, Ruste et al. also reported that patients with a high NLR had severe irAEs. This contradicts this present analysis’ finding of an association between an NLR of <4 and the development of irAEs. Our findings are supported by other works that found a higher frequency of irAEs in patients with a low NLR (22–25) and the fact that the presence of irAEs is consistently associated with better survival outcomes and a low NLR is a prognostic indicator and predictive marker of response to immunotherapy in advanced NSCLC (11, 26, 27). The NLR and/or SII have already been incorporated in prognostic scoring tools for these patients such as the NHS-Lung score, Lung Immune Prognostic Index (LIPI), and Lung Immune Prognostic score (LIPS) (10, 28–31). In addition to this, other works have highlighted the use of interleukin-6 (IL-6) and cytotoxic T-lymphocyte-associated antigen 4 (CTLA-4) levels and tumour burden to predict the occurrence of irAEs (32–34).

## Conclusions

The results of this retrospective analysis have shown that patients with irAEs had better survival outcomes. It has identified potential predictors of patients developing irAEs, including the NLR score, the SII score, the NHS-Lung score, disease response, and treatment discontinuation. The NHS-Lung score is an easy-to-use tool that can help predict not only prognoses in patients with advanced NSCLC on chemoimmunotherapy but also the likelihood of irAEs. The use of these scores may lead to a more proactive approach to identifying patients at risk of irAEs and therefore their prompt management, avoiding these irAEs progressing in severity.

## Data availability statement

The original contributions presented in the study are included in the article/**Supplementary Material**. Further inquiries can be directed to the corresponding author.

## Ethics statement

Ethical review and approval was not required for the study on human participants in accordance with the local legislation and institutional requirements. Written informed consent for participation was not required for this study in accordance with the national legislation and the institutional requirements.

## Author contributions

Conceptualisation: GB, AA, FG Methodology: GB, AS Software: GB, AS Validation; AS Formal analysis: GB, AS Investigation: JC, AC, CE, DP, CO, CC, SCh, SM. Resources: LM, SCa Data curation: PH, RB, CC, AC, AA, HM, GB Original draft: SA, GB Supervision: GB, CE, AA Project administration: LM, SCa All authors contributed to manuscript revision and approved the submitted version. FG and GB contributed equally as co-last authors.
