# Supplementary material for: Immunotherapy-related adverse events in real-world patients with advanced non-small cell lung cancer on chemoimmunotherapy: a Spinnaker study sub-analysis
Source: Front Oncol. 2023 May 31;13:1163768. doi: 10.3389/fonc.2023.1163768 (PMC10265987; doi:10.3389/fonc.2023.1163768)
Supplement: Supplementary file 1 [file DataSheet_1.docx]

**Supplementary Table 1: Subgroup analysis**

| **Subgroup** | **Value** | **No** | **irAE any** | | | **irAE G1-G2** | | | **irAE G3-G4** | | |
| --- | --- | --- | --- | --- | --- | --- | --- | --- | --- | --- | --- |
|  |  |  | **No.** | **%** | **p-value** | **No.** | **%** | **p-value** | **No.** | **%** | **p-value** |
| **NLR** | **> 4** | 164 | 59 | 36 | **0.0128** | 43 | 26 | **0.0175** | 25 | 15 | 0.8536 |
|  | **< 4** | 144 | 73 | 51 |  | 57 | 40 |  | 24 | 17 |  |
| **SII** | **> 1440** | 154 | 56 | 36 | **0.0287** | 41 | 27 | **0.0386** | 24 | 16 | 1 |
|  | **< 1440** | 154 | 76 | 49 |  | 59 | 38 |  | 25 | 16 |  |
| **Number of metastatic sites** | **> 3** | 103 | 39 | 38 | 0.2572 | 31 | 30 | 0.6165 | 10 | 10 | 0.0519 |
|  | **< 3** | 205 | 93 | 45 |  | 69 | 34 |  | 39 | 19 |  |
| **NHS Lung** | **0-1** | 224 | 108 | 48 | **0.0019**^b^ | 83 | 37 | **0.0076**^b^ | 40 | 18 | 0.1766^b^ |
|  | **2** | 84 | 24 | 29 |  | 17 | 20 |  | 9 | 11 |  |
| **Best response**^a^ | **CR/PR/SD** | 250 | 119 | 48 | **0.0011**^c^ | 88 | 35 | **0.0337**^c^ | 45 | 18 | **0.0388**^c^ |
|  | **PD** | 45 | 9 | 20 |  | 8 | 18 |  | 2 | 4 |  |
| **Discontinuation** | **Yes** | 72 | 57 | 79 | **<0.0001** | 31 | 43 | **0.0406** | 39 | 54 | **0.0001** |
|  | **No** | 236 | 75 | 32 |  | 69 | 29 |  | 10 | 4 |  |

Abbreviations: CR, complete response; irAE, immunotherapy-related adverse effects; NLR, neutrophil-to-lymphocyte ratio; No. Number; PD, progressive disease; PR, partial response; SD, stable disease; SII, systemic immune-inflammatory index; TPS, tumour proportion score;

a: 13 patients not assessable, b: NHS-Lung score 0-1 versus NHS-Lung score 2, c: CR, PR and SD versus PD

**Supplementary Table 2: Correlation analysis**

| **Variable** | **irAE any** | | **irAE G1-2** | | **irAE G3-4** | |
| --- | --- | --- | --- | --- | --- | --- |
|  | Correlation coefficient | p value | Correlation coefficient | p value | Correlation coefficient | p value |
| **NLR <4** | -0.148 | **0.009** | -0.142 | **0.013** | -0.0184 | 0.749 |
| **SII <1440** | -0.131 | **0.021** | -0.123 | **0.031** | -0.00749 | 0.896 |
| **NHS Lung** | -0.174 | **0.002** | -0.141 | **0.014** | -0.091 | 0.113 |
| **Best disease response** | -0.232 | **<0.001** | -0.178 | **0.002** | -0.104 | 0.076 |
| **Discontinuation** | 0.405 | **<0.001** | 0.122 | **0.034** | 0.577 | **<0.001** |
| Age | 0.0141 | 0.805 | 0.0306 | 0.593 | 0.0363 | 0.528 |
| **Gender** | -0.0962 | 0.092 | -0.062 | 0.281 | -0.0386 | 0.501 |
| **Smoking** | 0.0172 | 0.764 | 0.0305 | 0.596 | 0.0331 | 0.565 |
| **Histology** | -0.111 | 0.057 | -0.0927 | 0.113 | -0.0492 | 0.400 |
| **Stage** | -0.0591 | 0.321 | -0.0453 | 0.449 | -0.0959 | 0.109 |
| **Number of  metastatic sites** | -0.0715 | 0.211 | -0.0362 | 0.529 | -0.121 | **0.035** |
| **Brain metastases** | -0.00623 | 0.913 | -0.05 | 0.384 | 0.0301 | 0.600 |
| **Liver metastases** | -0.0375 | 0.512 | -0.0242 | 0.674 | -0.0532 | 0.355 |
| **ECOG PS** | 0.0724 | 0.205 | 0.0328 | 0.568 | 0.0407 | 0.479 |
| **BMI** | -0.0845 | 0.139 | -0.0857 | 0.135 | -0.0455 | 0.429 |
| **PD-L1 TPS** | 0.0105 | 0.857 | -0.00432 | 0.941 | 0.0241 | 0.681 |
| **Steroid use** | -0.00303 | 0.958 | -0.0269 | 0.640 | 0.0597 | 0.299 |
| **GCSF use** | 0.0619 | 0.278 | 0.0823 | 0.151 | -0.0108 | 0.851 |

Abbreviations: BMI, body mass index; ECOG PS, Eastern Cooperative Oncology Group Performance Status; GCSF, granulocyte colony-stimulating factor; irAE, immunotherapy-related adverse effects; NLR, neutrophil-to-lymphocyte ratio; No. Number; PD-L1, programmed cell death-ligand-1; PD, progressive disease; PR, partial response; SD, stable disease; SII, systemic immune-inflammatory index; TPS, tumour proportion score

**Supplementary Table 3: Cox regression analysis of overall survival according to type of immunotherapy-related adverse effects**

| **Variable** | **Hazard-ratio** | **95% confidence interval – low** | **95% confidence interval – high** | **P Value** |
| --- | --- | --- | --- | --- |
| **irAE single organ** | 0.53 | 0.38 | 0.73 | **<0.001** |
| **irAE multiple organ** | 0.53 | 0.30 | 0.95 | **0.032** |
| **Single organ**^a^**:** |  |  |  |  |
| **irAE skin** | 0.51 | 0.27 | 0.94 | **0.032** |
| **irAE colitis** | 0.61 | 0.33 | 1.13 | 0.117 |
| **irAE thyroid** | 0.33 | 0.15 | 0.76 | 0.009 |
| **irAE pneumonitis** | 0.65 | 0.34 | 1.25 | 0.198 |
| **irAE liver** | 0.51 | 0.22 | 1.16 | 0.109 |
| **irAE other** | 0.53 | 0.26 | 1.09 | 0.085 |

Abbreviations: irAE, immunotherapy-related adverse effects.

^a^Organ toxicity refers to single organ irAE occurring in at least 10 patients.

In bold significant P values.

**Supplementary Table 4: Cox regression analysis of progression-free survival according to type of immunotherapy-related adverse effects**

| **Cox-model (PFS)** | **Hazard-ratio** | **95% confidence interval – low** | **95% confidence interval – high** | **P Value** |
| --- | --- | --- | --- | --- |
| **irAE single organ** | 0.63 | 0.47 | 0.85 | **0.002** |
| **irAE multiple organ** | 0.61 | 0.37 | 1.01 | 0.056 |
| **Single organ**^a^**:** |  |  |  |  |
| **irAE skin** | 0.59 | 0.34 | 1.04 | 0.066 |
| **irAE colitis** | 0.83 | 0.49 | 1.42 | 0.499 |
| **irAE thyroid** | 0.48 | 0.24 | 0.94 | **0.032** |
| **irAE pneumonitis** | 0.67 | 0.38 | 1.18 | 0.163 |
| **irAE liver** | 0.65 | 0.32 | 1.34 | 0.246 |
| **irAE other** | 0.61 | 0.33 | 1.13 | 0.114 |

Abbreviations: irAE, immunotherapy-related adverse effects.

^a^Organ toxicity refers to single organ irAE occurring in at least 10 patients.

In bold significant P values.
